# T2-fluid attenuated inversion recovery mismatch in tumefactive multiple sclerosis

**DOI:** 10.1259/bjrcr.20220138

**Published:** 2023-02-07

**Authors:** Duc Le, Kelly Trinh, Nirav Das, Anderson H. Kuo

**Affiliations:** 1 Texas Tech University Health Sciences Center, Lubbock, Texas, United States; 2 Department of Radiology, Midland Memorial Hospital, Midland, Texas, United States; 3 Department of Radiology, University of Texas Health Science Center at San Antonio, San Antonio, Texas, United States

## Abstract

The T2-fluid attenuated inversion recovery (FLAIR) mismatch sign has been suggested as an imaging marker of isocitrate dehydrogenase-mutant 1p/19q non-codeleted gliomas with 100% specificity. Tumefactive demyelination is a common mimic of neoplasm that has led to unnecessary biopsies and even resections. We report a case of tumefactive multiple sclerosis in a 46-year-old male without prior symptomatic demyelinating episodes that demonstrates the T2-FLAIR mismatch sign. Our findings suggest the T2-FLAIR mismatch sign should not be used as a differential feature between glioma and tumefactive demyelination. Because typical isocitrate dehydrogenase-mutant 1p/19q non-codeleted gliomas typically do not demonstrate significant enhancement, such diagnosis should be reserved when post-contrast images are unavailable.

## Introduction

**Figure 1.**
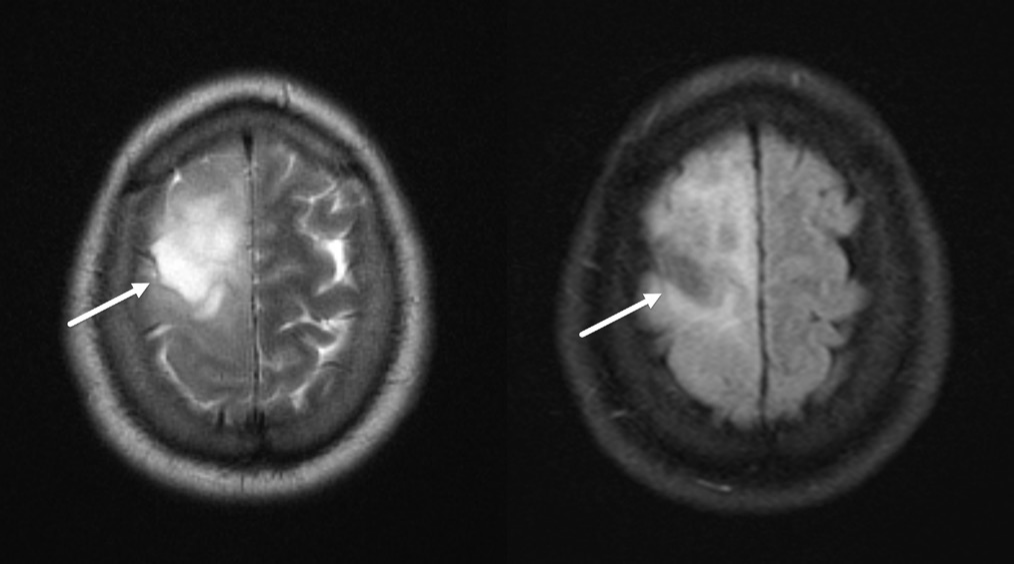
T2-FLAIR mismatch sign in IDH-mutant 1p/19q non-codeleted glioma. Axial T2 (left) and FLAIR (right) MR images demonstrate a large T2 hyperintense lesion in the right frontal lobe, which demonstrates FLAIR signal suppression (arrows), typical of the T2-FLAIR mismatch sign. FLAIR, fluid attenuated inversion recovery; IDH, isocitrate dehydrogenase.

In neuro-oncology, there is increasing role for molecular information due to its implication on histologic typing, treatment responsiveness, and overall prognosis. Accordingly, there is growing presence of genotypic profiles in the classification of central nervous system tumors in the more recent World Health Organization (WHO) classification schemes.^1^ In keeping with such trend, the T2-fluid attenuation inversion recovery (T2-FLAIR) mismatch sign has been recently described as a finding on MRI with near perfect specificity of isocitrate dehydrogenase (IDH) mutation and 1p/19q non-codeletion status of gliomas.^2^ The sign denotes complete/near-complete T2 hyperintense appearance of the glioma that suppresses on FLAIR images except for the peripheral rim, leading to mismatch of the T2 and FLAIR appearance (Figure 1). In a metanalysis of 10 cohorts across 8 studies, a pooled specificity of 100% was reported.^3^ However, the application of this imaging finding is at times questionable in clinical practice. At the initial imaging interpretation, the primary question is often not the molecular status of the glial neoplasm, but whether the observed lesion is a glioma. This subtle difference has anecdotally led to some diagnostic mishaps, as there is relative paucity of literature regarding the latter question and T2-FLAIR mismatch. In this report, a case of tumefactive multiple sclerosis that demonstrates T2-FLAIR mismatch is presented.

## Clinical presentation

A 46-year-old previously healthy male presented to the emergency department with a chief complaint of slowly worsening slurred speech for 10 days and right-sided facial droop and numbness that followed a few days later, discovered upon teeth brushing. The patient was previously seen at an outside facility, where a non-contrast CT of the head was performed. Per patient, the study revealed a “stroke,” but the images and report were not available. No other symptoms were reported. Common comorbidities associated with cerebrovascular accidents, such as diabetes mellitus, hypertension, and hyperlipidemia, were denied.

**Figure 2.**
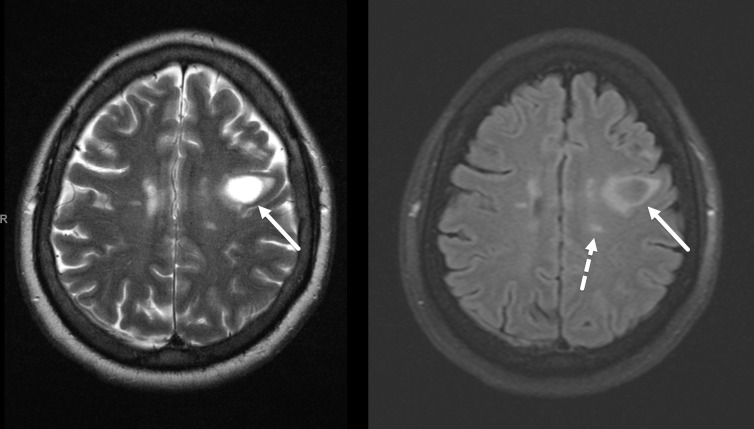
Apparent T2-FLAIR mismatch sign in a 46-year-old male with no significant past medical history. Axial T2 (left) and FLAIR (right) MR images demonstrate a 3 cm T2 hyperintense lesion in the left posterior frontal lobe (solid arrows). There is FLAIR signal suppression of the lesion, similar in appearance to the T2-FLAIR mismatch sign. Additionally, there are oval T2 hyperintense foci in the periventricular white matter in roughly perpendicular orientation to the ventricles (dashed arrow). FLAIR, fluid attenuated inversion recovery.

**Figure 3.**
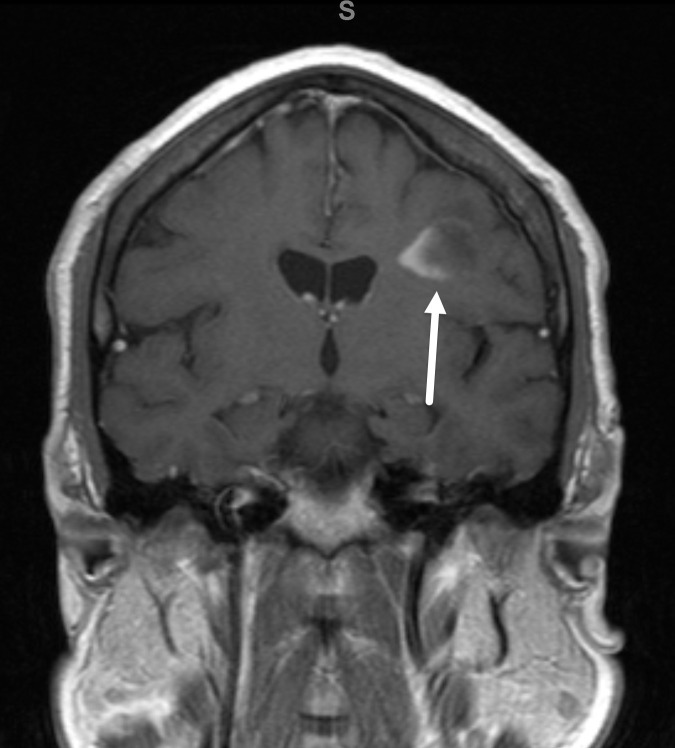
Incomplete rim enhancement of the left frontal lobe lesion. Post-contrast coronal T1 MR image through the lesion in Figure 2 demonstrates incomplete rim enhancement open to the cortical surface (arrow). This finding is typical of demyelination.

In-house MRI of the head was performed initially without contrast to confirm the infarction. No infarct was seen, but there was a 3 cm T1 hypointense, T2 hyperintense lesion in the left frontal lobe with mass effect/volume expansion (Figure 2, left). Signal suppression on FLAIR images was noted in similar description of the T2-FLAIR mismatch sign (Figure 2, right). Additionally, a few small T2 hyperintense foci were observed in the periventricular white matter in a slightly radiating pattern (Figure 2), reminiscent of multiple sclerosis. The patient was thus recalled for contrast enhanced imaging. On post-contrast images, there was classic incomplete rim enhancing of the left frontal lobe lesion with the open rim toward the cortical surface (Figure 3). The impression of tumefactive demyelination/multiple sclerosis was rendered with recommendation for cerebrospinal fluid (CSF) analysis.

**Figure 4.**
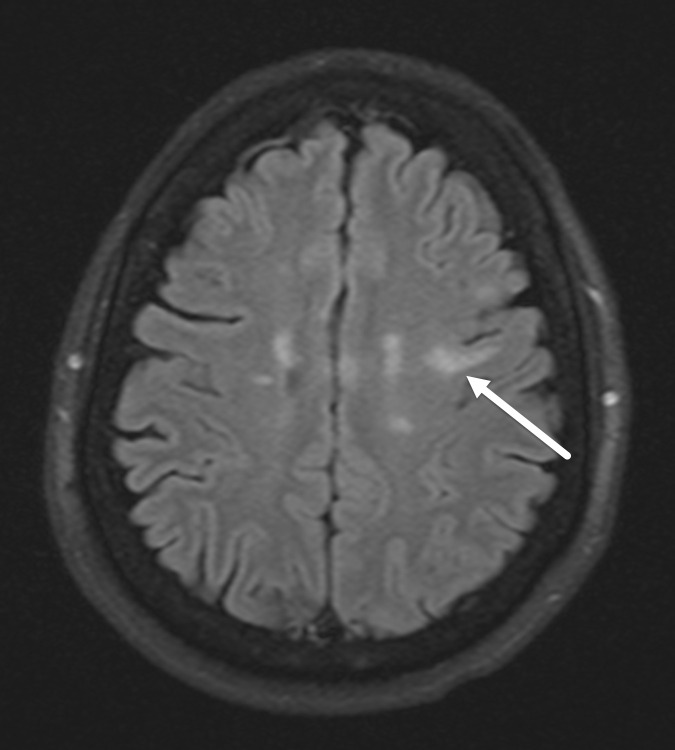
Partial resolution of the left frontal lobe lesion on follow-up. Axial FLAIR image demonstrates partial resolution of the left frontal lobe lesion at 4 month follow-up, formally excluding neoplasm as a differential. FLAIR, fluid attenuated inversion recovery.

Lumbar puncture was successfully performed with normal opening pressure, yielding clear CSF. Laboratory analysis revealed positive oligoclonal bands suggestive of multiple sclerosis but was otherwise unremarkable. Presumed active demyelination was diagnosed, and intravenous methylprednisolone (Solu-Medrol, 1 g/day) was initiated for 5 days. Significant symptomatic improvement was noted towards the end of the intravenous therapy, and the patient was discharged on an oral steroid taper for 1 week. Follow-up MRI at 4 months demonstrated resolution of the previously seen left frontal lobe mass and T2-FLAIR mismatch sign with patchy area of residual T2 signal alteration (Figure 4).

## Discussion

Despite the very high specificity of T2-FLAIR mismatch sign for the diagnosis of IDH-mutant 1p/19q non-codeleted glioma, this imaging feature should be applied in caution due to the nature of the available literature. The specificity data of the mismatch sign originate nearly entirely from glioma cohorts. This selection bias suggests that the mismatch sign only specifically categorizes lesions that have otherwise been diagnosed as (lower grade) gliomas, at least by available data. While detection of such sign in a parenchymal lesion does give some credence to a potential glial neoplasm, the true specificity is likely lower when nonglial lesions are included. In this report, a case of tumefactive demyelination serves as an important reminder of such scenario.

Indeed, a comparable T2-FLAIR sign has previously been reported for dysembryoplastic neuroepithelial tumors by the name of “bright rim sign.”^4^ Parenchymal cysts or other cystic lesions (such as pleomorphic xanthoastrocytoma^5^) can also demonstrate FLAIR signal suppression. Mechanistically, the FLAIR sequence utilizes an inversion pulse to suppress the normally bright signal of simple fluid/CSF. Consequently, any T2 hyperintense structure with similar longitudinal relaxation curve to CSF may demonstrate FLAIR suppression. In IDH-mutant 1p19q non-deleted gliomas, confluent microcystic spaces are thought to be responsible for the FLAIR signal suppression (although not reaching statistical significance in the proposed study).^2^ In cystic lesions, the similarity of the fluid content to CSF leads to suppression of its signal on FLAIR images.

**Figure 5.**
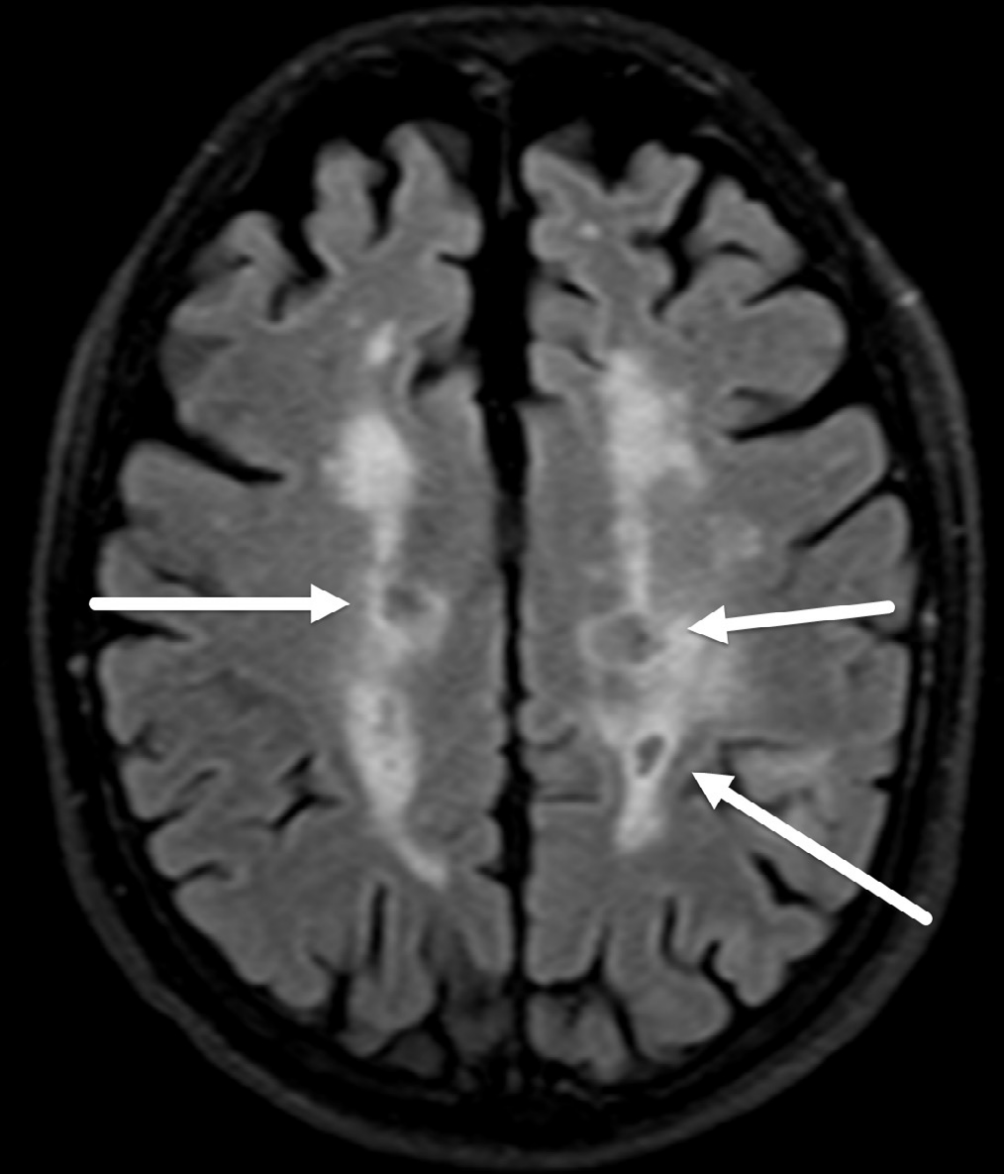
FLAIR signal suppression in T1 black holes in high burden multiple sclerosis. Axial FLAIR MR image shows areas of FLAIR signal suppression in the periventricular lesions (arrows) in a patient with known multiple sclerosis, corresponding to T1 black holes, a finding often seen in patients with severe disease burden. FLAIR, fluid attenuated inversion recovery.

In tumefactive demyelination, severe myelin loss, necrosis, and reactive gliosis are known to occur centrally with hypercellularity from Creutzfeldt cells (reactive astrocytes), myeline protein-laden macrophages, variable lymphocytic infiltrates, and relative axonal sparing.^6–8^ On imaging, there is frequently resultant mass effect (45% of the cases) and edema (77%), which is often moderate to marked (41%).^6^ These combined effects are thought to allow for the occasional suppression of FLAIR signal as in this case, although not methodologically proven. Several advanced MRI techniques are under ongoing investigation for the evaluation of demyelinating disease (such as myelin water imaging and quantitative susceptibility mapping) that may serve as helpful differentiating markers^9,10^; sadly their use is not yet clinically mature. It should be also remembered that T1 black holes are commonly seen in patients with high burden multiple sclerosis where plaques of severe myelin loss, axonal injury, and matrix destruction are present^11,12^ and with the more recently described slowly expanding lesions where progressive decline in T1 intensity suggests ongoing neuro-axonal damage.^13,14^ These T1 black holes are also typically T2 hyperintense and hypointense on FLAIR images (Figure 5).

Since the original description of the T2-FLAIR mismatch sign, Jain et al have proposed additional criteria to better qualify the applicability of this finding.^15^ Exclusion of cystic or necrotic components, excluding lesions with significant enhancement, allowing for some inhomogeneity of FLAIR suppression, and avoidance of calling such sign in pediatric patients are thought to help improve accuracy. However, large-scale studies are needed to better validate these proposed restrictions, and interobserver agreeability requires further study. Technical factors (such as exact inversion time, scanner strength, sequence type, and pre- *vs* post-contrast T2 FLAIR acquisition) may also influence appearance of this finding. In this case, the post-contrast images are suggestive of the diagnosis once obtained. However, the presented case does raise concern for the applicability of the T2-FLAIR mismatch sign on non-contrast studies.

## Learning points

1. When other MRI features of a mass lesion are consistent with low-grade glioma, T2-FLAIR mismatch sign has near perfect specificity for IDH-mutation and 1p/19q co-deletion status.
2. The T2-FLAIR mismatch sign can be seen in tumefactive demyelination and non-glial neoplasms, warranting caution in its interpretation in isolation before the lesion is fully characterized.
3. The mismatch sign should not be applied to cystic or necrotic components of tumors or pediatric patients. Lesions with significant enhancement (and thus non-contrast studies) should also be viewed with suspicion.
4. The T2-FLAIR mismatch sign has not yet been studied in detail in higher grade gliomas, and its role is less certain.
